# Stability of Alkanethiol Self-Assembled Monolayers of Varied Chain Lengths for Area-Selective Atomic Layer Deposition

**DOI:** 10.1021/acs.langmuir.6c01484

**Published:** 2026-07-15

**Authors:** Henry Price, Vamseedhara Vemuri, Michelle M. Paquette, Nicholas C. Strandwitz

**Affiliations:** † Department of Materials Science and Engineering and Institute for Functional Materials and Devices, 1687 Lehigh University, Bethlehem, Pennsylvania 18015, United States; ‡ Missouri Institute for Defense & Energy and Division of Energy, Matter & Systems, 12273 University of Missouri Kansas City, Kansas City, Missouri 64110, United States

## Abstract

Alkanethiol (CH_3_(CH_2_)_*n*−1_SH) self-assembled monolayers (SAMs) are a common blocking layer in area-selective atomic layer deposition (AS-ALD), yet the stability and selectivity of this class of SAMs under different deposition conditions have not been fully explored. Here, alkanethiol SAMs of varying carbon chain lengths (*n* = 6, 12, and 18) were tested for their ability to prevent ALD of alumina and hafnia on copper surfaces using trimethylaluminum (TMA) and tetrakis­(dimethylamino)­hafnium (TDMAH) as metal precursors at temperatures from 100 to 180 °C. Additionally, monolayer stability was evaluated by exposing the SAMs to isolated ALD processing variables—elevated temperature, metal precursor vapor exposure, and water vapor exposure. Results showed that selectivity was higher for hafnia growth and increased with chain length, though no clear trend as a function of chain length was observed for alumina deposition. SAMs of all chain lengths showed clear degradation via loss of sulfur with TMA exposure, suggesting that drops in selectivity are due to penetration of the smaller, more reactive TMA molecule through the SAM to reach surface-bound sulfur, resulting in SAM desorption. In general, alkanethiol SAMs display unique blocking behavior that is dependent on carbon chain length, temperature, and metal precursor size and reactivity, with the highest selectivity displayed by a long, ordered SAM (*n* = 18) against a larger, less reactive precursor (TDMAH).

## Graphical abstract

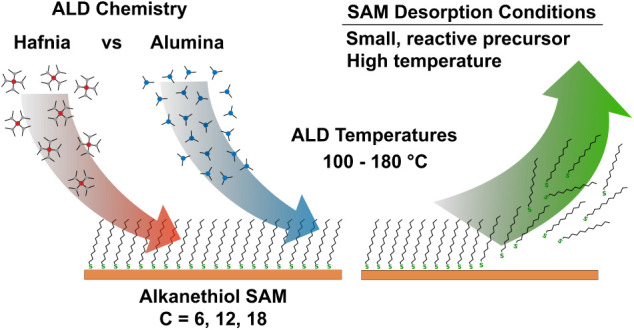

## Introduction

As semiconductor fabrication advances to nanometer-scale feature sizes, increasingly precise spatial control over thin film deposition and placement on patterned surfaces is desired. Techniques such as atomic layer deposition (ALD) enable atomic-scale control of thickness, but additional subtractive etching is required to control the film pattern.^1,2^ Sequential growth and etching steps are time-intensive and costly, and at the nanoscale, these conventional top-down fabrication methods can encounter challenges like pattern misalignment and edge placement errors.^1–3^ Area-selective deposition (ASD), a bottom-up method for spatial control, could help to eliminate the aforementioned challenges and reduce the number of processing steps.^4^ Unlike lithography or other top-down techniques, ASD uses only the inherent chemical, structural, or physical properties of the surface and reactants to spatially control deposition.^1^

One way to achieve spatial selectivity is by leveraging differences in the chemical reactivity of the surface to passivate certain areas.^1^ Often, a “blocking layer” is used, which is a film or monolayer that preferentially bonds only to certain surface chemistries of a substrate and inhibits deposition. Organic self-assembled monolayers (SAMs) are often used as blocking layers.^5^ Each molecule in a SAM contains a headgroup that binds to the substrate, a tail group that determines the monolayer surface properties, and a backbone in between the two that influences the packing of the surface-bound molecules. The blocking ability of SAMs can be influenced by each part of the molecular structure in different ways, allowing for a variety of pathways for improving their performance. One class of SAMs, *n*-alkanethiols, has been extensively examined in the context of ASD.^6–12^ Alkanethiols (CH_3_(CH_2_)_*n*−1_SH) are made up of a sulfur headgroup, carbon chain backbone, and methyl tail group. The sulfur headgroup preferentially adsorbs onto a metal surface to form a sulfur–metal bond, and the carbon chains self-orient tilted a few degrees from surface normal,^13,14^ forming a densely packed molecular monolayer that has been shown to have passivating properties, including protection against surface metal oxidation.^15^ In ASD applications, the terminal hydrophobic methyl group has been shown to play an important role in preventing ALD growth on the SAM, by blocking precursor access to the underlying layer and resisting reaction itself with most ALD precursors.^16,17^

Alkanethiol SAMs, however, are not without limitations to their ability to prevent film growth, as growth on these SAMs eventually occurs after some number of ALD cycles. Additionally, their stability and structure are temperature dependent.^18^ At high temperatures (150–160 °C), monolayers of alkanethiols on copper have been shown to desorb, with the thiolate group oxidizing to a sulfonate group.^18–20^ While desorption represents the most detrimental blocking layer breakdown mechanism, SAMs can also become disordered without fully desorbing, creating discontinuities in the monolayer, which can then act as nucleation sites for film growth.^21^ Many film growth processes take place at temperatures that may fall at or above the temperature of SAM desorption/destabilization, so to expand the stability window for blocking-layer-enabled ASD, a clearer understanding and optimization of blocking layer stability is desired.

Alkanethiol SAM behavior, including phase behavior, has been shown to be chain-length dependent,^22^ and it has been well established that backbone chain length has a strong influence on the packing density and degree of order of the SAM.^23,24^ Alkanethiol SAMs have been shown to increase in hydrophobicity with chain length, which is attributed to denser packing.^11,25^ SAMs with longer chains (*n* > 16) have been shown to be superior to those with shorter chains (*n* < 12) at preventing corrosion of copper due to increased interchain van der Waals interaction, and thus higher crystallinity.^26^ Similar behavior of increasing crystallinity with chain length has been shown for alkanethiol SAMs on gold surfaces.^27,28^ Simulation studies of different chain lengths in islands of alkanethiols showed that fewer molecules were needed for ordered islands when chain lengths were longer, supporting conclusions that longer chain lengths increase crystallinity.^29^ Other studies have shown decreased reactivity or degradation of alkanethiol SAMs on Cu by atomic hydrogen with increased chain length.^30,31^ More directly related to ASD, hydrophobicity has also been shown to be related to the blocking ability of the monolayers. In one study, blocking of hafnia deposition approximately tracked with the water contact angle of those monolayers, and alkoxysilane monolayers with alkyl chain lengths of 12 and greater completely blocked deposition.^32^ However, other work has shown that water contact angle alone is not an effective predictor of blocking ability.^33^ Additionally, chain length has shown a significant impact on the ability to block alumina, with a 12-carbon alkanethiol reducing alumina deposition to less than half that observed with a 2-carbon alkanethiol.^6^ In general, from existing literature on alkanethiols and other SAM species, longer chains are expected to hinder film growth better than shorter chains. However, the behavior of different chain-length alkanethiol SAMs under ALD conditions and the associated mechanisms of destabilization have yet to be tested in a systematic study. This work investigates the performance of alkanethiol SAMs with three different chain lengths (*n* = 6, 12, and 18) as blocking layers on copper for AS-ALD of alumina and hafnia at 100–180 °C using common ALD precursors. Alkanethiol SAMs were selected because of their widespread use as blocking layers and the simplicity of their tail structure, making them useful as a model system. Furthermore, to elucidate the cause and potential mechanism of SAM destabilization, the alkanethiol layers were exposed to isolated ALD process variables, revealing important insights into their stability and selectivity.

## Experimental Section

### SAM Growth

All alkanethiol SAMs were grown on copper-coated silicon wafers from 10 mM solutions in ethanol. Stock solutions of hexanethiol, dodecanethiol, and octadecanethiol were prepared by diluting pure alkanethiols (Sigma-Aldrich) with reagent-grade ethanol. Substrates were Si (100) wafers coated with ∼200 nm of Cu using electron-beam evaporation. To clean the Cu surface immediately prior to SAM formation, the wafers were rinsed with ethanol followed by water and then dried under a nitrogen gas stream. The wafers were then subjected to UV ozone exposure for 3 min (Jelight), rinsed again sequentially with ethanol, then water, then ethanol, dried with nitrogen gas, and finally left to sit for 1 h in ambient air, as controlled oxidation has been shown to increase SAM quality and blocking ability.^9^ To form the SAM films, wafers were immersed in 10 mL of the ethanolic thiol solutions and placed on a hot plate set to 40 °C while covered with parafilm and an inverted beaker. Samples were immersed for 1 h,^34^ which is expected to be sufficient time to form a uniform monolayer.^35,36^ During this time, any wafers on which SAMs were not being grown were placed in a desiccator chamber to limit differences in air exposure between coated and bare samples. After immersion, samples were rinsed 3 times with ethanol and dried with a nitrogen gas stream.

### Film Deposition

Atomic layer deposition was conducted using an Ultratech Cambridge Nanotech Savannah 100 ALD system. For the growth of alumina and hafnia films, the precursors trimethylaluminum (TMA, 99%, STREM Chemicals) and tetrakis­(dimethylamido)hafnium (TDMAH, 99%, STREM Chemicals) were used. The TMA and water precursors were held at room temperature, and the TDMAH precursor supply was heated to 80 °C. A vapor-draw delivery system was used for all precursors. Before all runs, a wait time of 300 s with samples loaded under N_2_ (g) flow was used. Research-grade N_2_ (99.9999%, Airgas) was used as the carrier gas at a flow rate of 20 sccm, resulting in a deposition pressure of approximately 0.1 Torr. Precursor pulse times of 0.025 s for TMA, 0.1 s for TDMAH, and 0.08 s for water were used, with a 20 s purge time between precursor pulses with continuous N_2_ (g) flow. During deposition, the reactor walls and sample stage were heated to various temperatures as described below. The precursor delivery line, precursor manifold, and stop valve separating the vacuum pump from the sample chamber were all heated to 150 °C for all depositions. We note that we are within the ALD temperature windows for the ALD chemistries selected and that no nucleation delays were detected on bare Cu or bare Si surfaces with native oxide.

In some cases, isolated ALD process steps were conducted on Cu and alkanethiol-functionalized Cu surfaces. In these cases, samples were placed in the ALD chamber and either exposed to N_2_ flow at temperature, N_2_ flow at temperature with H_2_O exposure, or N_2_ flow at temperature with metal precursor exposure. Finally, growth per cycle (GPC) values for the TMA- and TDMAH-based ALD chemistries ranged from 0.98 to 0.99 Å/cycle and 1.0–1.2 Å/cycle, respectively, which are comparable to values in the literature.^37,38^

### Characterization

To evaluate the hydrophobicity of the Cu and SAM surfaces, water contact angle (WCA) data were collected with a Dataphysics OCA 20 Plus goniometer with SCA 20 software. Two microliters of water were used for each measurement, and the needle-in-drop method for static droplets was used.^39^ ImageJ software^40^ with the contact angle plugin was used to measure the water contact angle, and an average of the left and right contact angles was used for each droplet. Data from at least four droplets were collected for each sample.

Atomic composition data were collected using X-ray photoelectron spectroscopy (XPS) acquired with a custom-built SPECS XPS instrument using an Al K-α source with a photon energy of 1486.6 eV and a PHOIBOS 150 detector. The pass energy for survey scans was 70 eV, and the pass energy for core-level scans was 20 eV. Following data collection, the core-level peaks (C 1s, O 1s, S 2p, Cu 3p/Al 2p, Cu 3s/Al 2s, Si 2p, and Hf 4f) were analyzed using CasaXPS software. Binding energy correction was achieved by calibrating the C 1s peak to 284.8 eV and applying the correction to all core-level peaks for the sample. Individual core-level peaks were fit using a Gaussian/Lorentzian sum formula with a baseline aligned to the spectral background and adjusted to minimize the standard deviation. For split peaks, known energy differences between peaks were obtained from the online Thermo Fisher Scientific XPS database^41^ and applied as position constraints for peak fitting. Relative sensitivity factors (RSFs) were used from the Scofield library. Quantification of alumina or hafnia deposition was performed by determining the atomic percentage of Al or Hf relative to the total metal signal, given by Al/(Al + Cu) or Hf/(Hf + Cu). Quantification was done using the Al 2s and Cu 3s XP spectra for Al-based ALD and the Hf 4f and Cu 3s XP spectra for Hf-based ALD. This quantification was further used for selectivity calculations.

## Results and Discussion

Self-assembled monolayers of hexanethiol (C6), dodecanethiol (C12), and octadecanethiol (C18) were prepared from ethanol solutions on copper-coated silicon substrates. The hydrophobicity of the three SAMs was characterized by water contact angle (WCA) measurements as an indicator of monolayer uniformity. Average WCA values were 99.2 degrees (SD, standard deviation = 4.8) for C6, 113.2 degrees (SD = 3.7) for C12, and 122.3 degrees (SD = 2.3) for C18, confirming that the resulting SAMs were hydrophobic. In contrast, blank oxidized copper surfaces displayed an average water contact angle of 52.4 degrees (SD = 3.4), similar to WCA values for acetic acid-treated copper surfaces, an analogous copper oxide surface.^8^ The observed trend of increasing WCA with increasing SAM chain length is consistent with relationships established in the literature,^11^ which suggests that a longer chain length results in a more densely packed SAM structure due to the increased van der Waals interactions between chains.^32^ Furthermore, well-ordered alkanethiol SAMs have been shown to display higher WCA values than less ordered ones, as ordering creates a uniform SAM surface of the highly hydrophobic methyl tail group.^9,25,42^ Hydrophobicity is an important metric for blocking layer SAMs because densely packed and hydrophobic SAMs have been shown to be more effective at preventing ALD.^32^

The blocking ability of these SAMs toward 15 ALD cycles of alumina (TMA + H_2_O) or hafnia (TDMAH + H_2_O) was evaluated by XPS-quantified atomic percentage fractions of Al or Hf, normalized to the Cu substrate signal. Each of the alkanethiol SAM chain lengths (C6, C12, C18) was tested at deposition temperatures of 100 °C, 120 °C, 140 °C, 160 °C, and 180 °C (Figure 1). Note that a chain length of 0 indicates a SAM-free Cu surface (otherwise prepared identically to the SAM-functionalized surfaces). Different SAM blocking behavior for hafnia vs alumina was observed. For hafnia deposition at all temperatures, there appeared to be minimal difference between the SAM chain lengths in blocking at 15 cycles, with hafnia deposition nearly completely blocked for all combinations tested (Figure 1a). However, there did appear to be temperature and chain length dependence of the blocking ability toward alumina (Figure 1b). In this case, the three chain lengths displayed similar blocking efficacy up to temperatures of 180 °C, at which point a higher aluminum percentage [Al/(Al + Cu) > 0.3] was observed on the C6 SAM. This deposition presumably resulted from a specific characteristic of the C6 SAM that perhaps became disordered or desorbed at this high temperature, thereby allowing alumina nucleation, as further evidenced below. Hafnia deposition at 180 °C did not show any enhanced nucleation for any SAM length relative to lower temperatures, revealing differences in blocking ability toward the different Al and Hf precursor chemistries.

**Figure 1:**
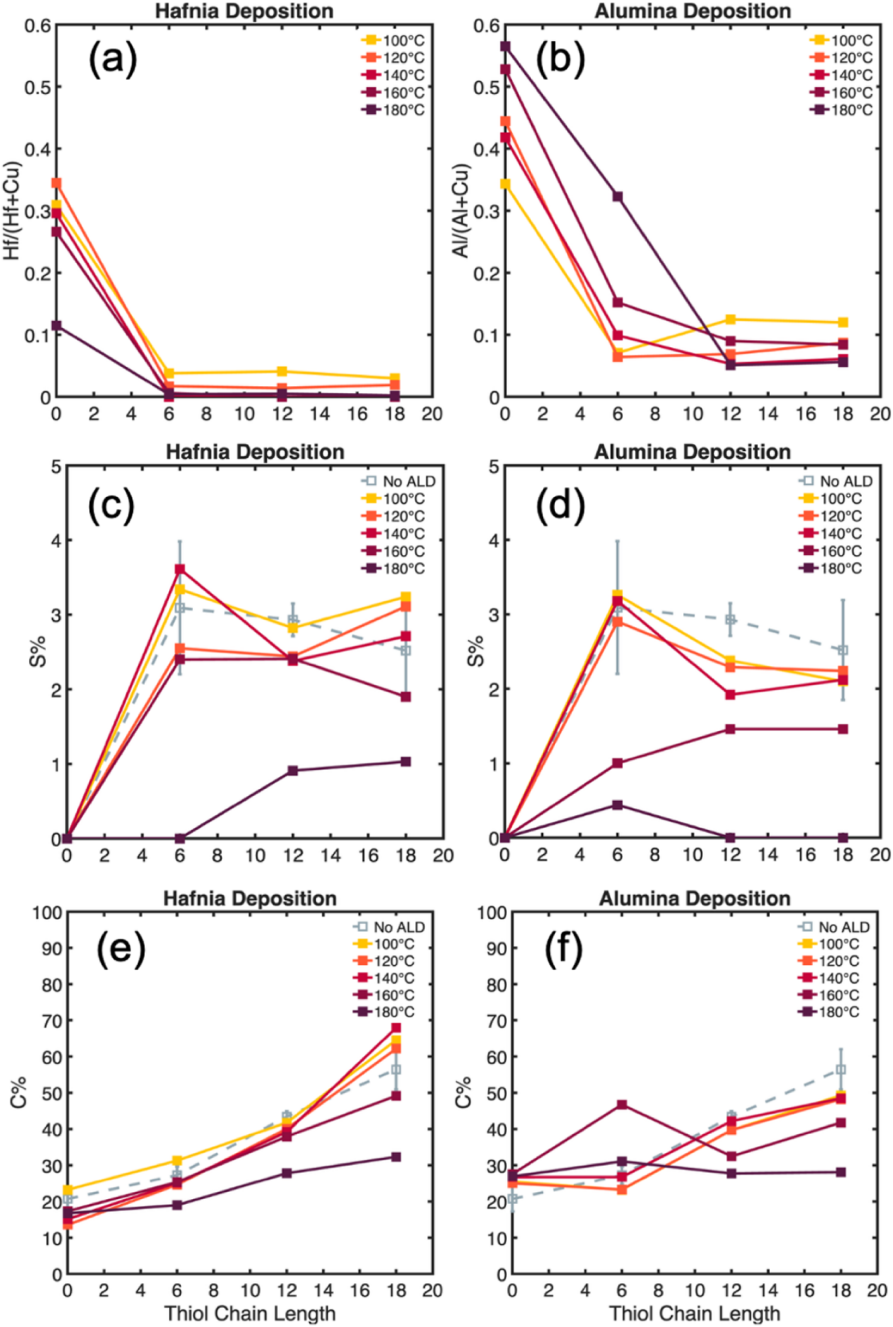
Hf (a) and Al (b) atomic percentage ratios for 15 ALD cycles on bare copper (chain length = 0) and Cu coated with alkanethiol SAMs of different chain lengths. Sulfur atomic percentages for the hafnia- (c) and alumina-coated (d) samples. Carbon atomic percentages for the hafnia- (e) and alumina-coated (f) samples.

Each alkanethiol molecule contains a sulfur headgroup that chemisorbs to the copper substrate during SAM formation (Figure S1). It is primarily the stability of this S–Cu bond that controls the ability of the SAM to remain adhered to the surface.^43^ Sulfur atomic concentrations were quantified to give insight into the impact of the ALD process on the SAM chemistry. For hafnia deposition at temperatures of 100 to 160 °C (Figure 1c), the sulfur percentage after 15 cycles of ALD remained within 1% of the sulfur percentages for the SAMs that were not subjected to ALD. However, at a deposition temperature of 180 °C, the sulfur percentage was significantly reduced as compared to the ALD-free case, from 3.1 to 0% for the C6 SAM, 2.9 to 0.9% for the C12 SAM, and 2.5 to 1.0% for the C18 SAM. Similar trends were observed in samples that received 15 cycles of alumina ALD, but with sulfur percentages showing significant drops at 160 °C, with a near complete absence of sulfur at 180 °C for all SAM chain lengths (Figure 1d). For only 15 cycles of ALD, the time spent at the deposition temperature is approximately 15 min (with a 300 s stabilization and two 20 s purge times per cycle, deposition time results in approximately 1 min per cycle). Alkanethiols have been shown to desorb after only 10 min of annealing at 160 °C,^19^ so 15 min may be sufficient for significant desorption to occur. We note that the lack of an XPS sulfur signal in these samples was not due to an attenuation of S 2p photoelectrons by the SAM, because this effect would be observed in the “no ALD” case, and photoelectron escape depths are 6–9 nm, greater than the maximum SAM thickness of ∼2.5 nm.^44,45^ Furthermore, attenuation by any ALD layer is also unlikely because very little film (∼1.5 nm at most for 15 cycles) is deposited in all cases, with the possible exception of the alumina deposition at 180 °C, and the underlying Cu is still clearly observed in all samples in Figure 1. Therefore, these low sulfur percentages at higher temperatures can tentatively be attributed to the loss of sulfur via SAM desorption or decomposition rather than coverage by additional material. However, future studies would benefit from replicating these experiments, as each sulfur percentage data point in this study represents only one measurement.

To clarify the fate of the SAM carbon species during ALD, as well as to probe the SAM alkyl chain structural integrity, total carbon percentages were also recorded from XPS analyses (Figure 1e, 1f). Total carbon included signals from the SAM alkyl chains as well as surface adventitious carbon, the presence of which was confirmed by nonzero carbon percentage values (an average of 20.7%) for blank copper samples with neither SAM nor ALD. Carbon percentages for samples with and without SAMs subjected to ALD were compared. Between SAMs of differing chain lengths (without ALD), there was a stepwise increase in average carbon percentages: 27.3%, 43.4%, and 56.5% for C6, C12, and C18 SAMs, respectively, aligning with the stepwise increase in the number of carbons per SAM alkyl chain. Hafnia deposition at temperatures of 100 °C through 160 °C did not appear to significantly alter carbon content from SAMs without ALD. However, at 180 °C, carbon content was reduced for all three chain lengths to 19.0%, 27.8%, and 32.3% for C6, C12, and C18, respectively (Figure 1e). As sulfur percentages for hafnia deposition also showed significant decreases at 180 °C, it is likely that whole alkanethiol molecules were desorbing under these conditions. Alumina deposition displayed slightly different temperature-dependent changes in carbon percentages (Figure 1f), most notably for temperatures of 160 and 180 °C. The high carbon content for C6 at 160 °C was most likely caused by anomalous adventitious carbon, as C12 and C18 displayed decreases from the samples without ALD. At 180 °C, carbon percentages for all three chain lengths (31.1%, 27.8%, and 28.1% for C6, C12, and C18, respectively) were similar to those of samples without a SAM (26.9%) that contained only adventitious carbon. This substantial decrease in carbon, alongside the observed decrease in sulfur content, further supports the hypothesis of temperature-dependent SAM desorption. It is not a given, however, that the remaining carbon signal is entirely adventitious carbon, and it is possible that in addition to desorption, a SAM fragmentation mechanism could leave behind alkyl chain fragments at the surface. Importantly, at 180 °C, both hafnia and alumina deposition were still inhibited (Figure 1a, 1b), even as SAM desorption was evidenced by XP spectra. It is possible that the kinetics of the competing deposition and desorption processes were such that, although ALD was initially inhibited by the presence of the blocking layers, a more gradual desorption of the SAMs still ultimately occurred. In addition, the presence of SAM fragments could contribute to growth inhibition. In either case, it is expected that increased ALD cycles would result in uninhibited deposition.

The C6, C12, and C18 alkanethiol SAMs were further tested for their ability to block ALD of alumina and hafnia at 15, 40, 50, and 60 cycles. These experiments were conducted at chamber temperatures of 140 and 180 °C. The selectivity (*S*) was quantified using the formula proposed by Gladfelter,^46^

$$S=\frac{R_{gs}-R_{ns}}{R_{gs}+R_{ns}}$$

where *R*_gs_ refers to the atomic ratio of the growth species, Al/(Al + Cu) for example, on the desired *growth surface*, in this case a blank Si coupon.^1,8^ *R*_ns_ refers to that same ratio but for the *nongrowth surface*, in this case the SAM film on a Cu surface. *S* values of 1 indicate complete blocking on the nongrowth surface. Using this metric, blocking layer and ALD chemistry combinations can be directly compared with each other and with results from the literature after a given number of ALD cycles.

Selectivity against hafnia growth at 140 °C showed little variation between SAM chain lengths, with all displaying moderate-to-high selectivity (Figure 2a). All chain lengths exhibited decreasing selectivity as ALD cycles increased, dropping to nearly 0.6 at 60 ALD cycles. However, at 180 °C, a clear correlation between hafnia selectivity and chain length emerged. For the same number of ALD cycles, the longer the chain length of the SAM, the higher the hafnia selectivity value (Figure 2b). The C18 SAM even displayed selectivity greater than 0.8 through 50 cycles, though it dropped to nearly 0.2 at 60 cycles. More generally, the difference in selectivity between deposition temperatures helps elucidate the upper limit of the optimal temperature range for each combination of ALD and SAM chain length. For hafnia deposition, selectivity remained high at 140 °C, while generally displaying lower values at 180 °C (Figure 2b), suggesting the upper temperature limit was somewhere between 140 and 180 °C. If the cause of lower selectivity was SAM degradation, it can be hypothesized that this limit lies somewhere above 160 °C, as sulfur percentage data for 15 cycles of hafnia deposition only showed a decrease at 180 °C, remaining high at 160 °C (Figure 1c). Such an upper bound could also be chain-length dependent, as sulfur content was zero for the C6 SAM at 180 °C but nonzero for C12 and C18 (Figure 1c). Finally, the lack of Hf deposition at 15 cycles at 180 °C (Figures 1a and 2b) may be explained by a slow rate of removal of the C6 SAM over approximately the first 15 cycles, as there was a rapid decrease in selectivity at higher cycle numbers, from ∼1.0 to nearly 0.25 from 15 to 40 ALD cycles.

**Figure 2:**
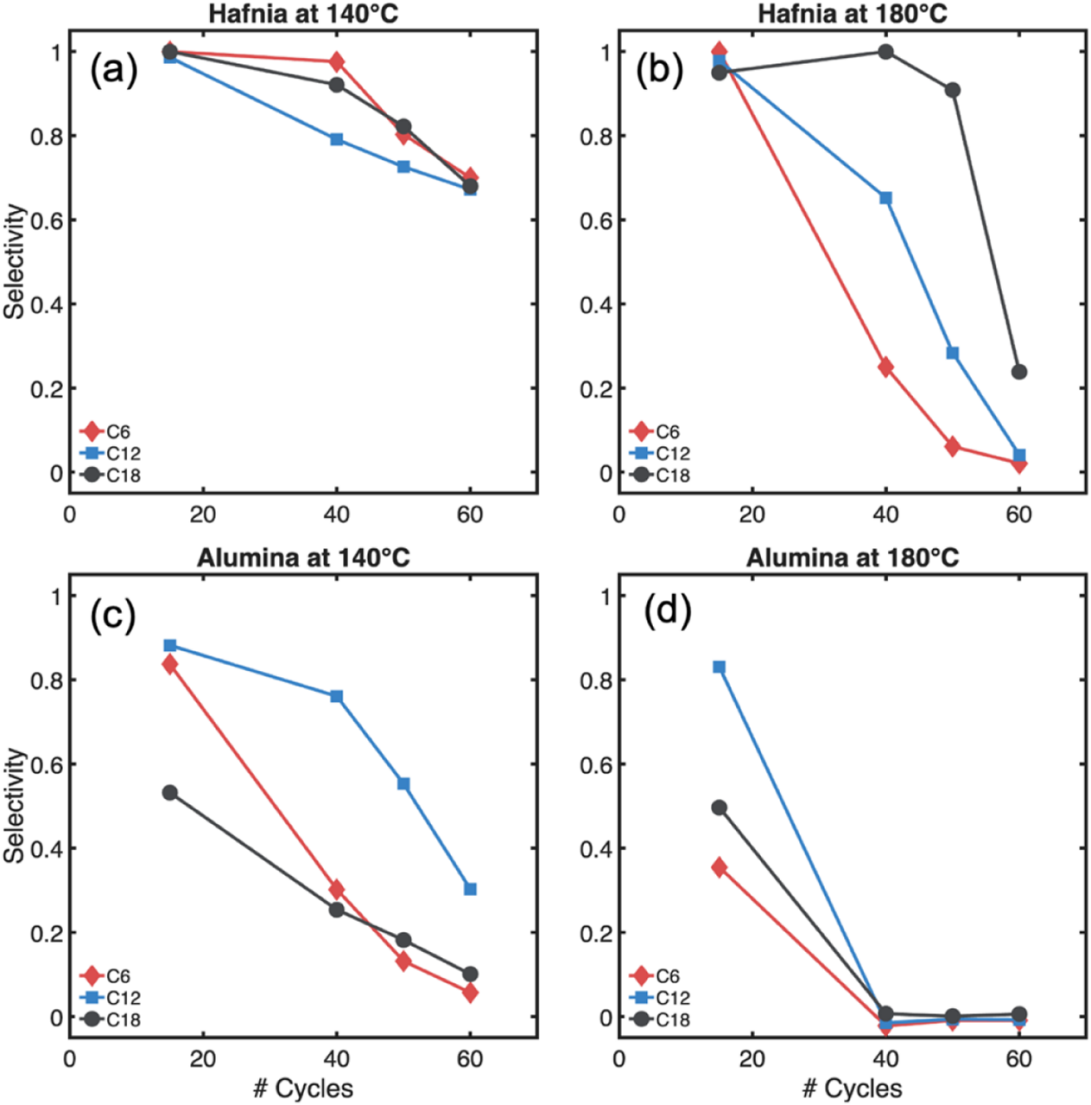
Selectivity data for alkanethiol SAMs with carbon chain lengths of 6, 12, and 18 for the growth of hafnia at 140 °C (a) and 180 °C (b), and for alumina at 140 °C (c) and 180 °C (d). Selectivity was calculated using SAMs on Cu as the nongrowth surface and Si as the growth surface.

Unlike hafnia, selectivity against alumina deposition displayed no clear correlation with chain length. At a deposition temperature of 140 °C, C6 and C18 SAMs exhibited similar selectivity at ≥40 cycles, while the C12 SAM showed higher values; however, the selectivity for all SAMs decreased with increasing ALD cycles (Figure 2c). At 180 °C, the C12 SAM again displayed the highest selectivity at 15 cycles of ALD, but beyond 40 cycles, all SAM chain lengths showed low selectivity, indicating no alumina blocking ability (Figure 2d). These results can be compared to the sulfur atomic percentage data for 15 cycles of alumina deposition where sulfur content was lower for both 160 and 180 °C (Figure 1d), supporting the theory that the SAMs are destabilized at those temperatures, thus decreasing selectivity. However, such a decrease in selectivity does not inherently indicate that SAMs are desorbed—deposition may have occurred at defects in the SAM without desorption. From these data, the upper temperature limit for these SAMs for preventing alumina growth is most likely lower than the upper temperature limit for hafnia. To make more concrete conclusions, future studies would benefit from testing smaller increments of cycle numbers, particularly for deposition of alumina between 15 and 40 cycles, to attempt to capture the selectivity drop. Furthermore, also testing selectivity at more granular temperature increments would help in specifying these temperature limits. In general, these SAMs were more resistant to nucleation of hafnia than alumina at both deposition temperatures, though selectivity was lower at 180 °C than 140 °C for both deposition chemistries. Additionally, the emergence of chain-length-dependent selectivity only at higher temperatures implies that selectivity is impacted by monolayer disordering, decomposition, or desorption, and that each chain length displayed a unique temperature range for each deposition chemistry where it could most effectively passivate the surface.

Different selectivity values for the deposition of alumina and hafnia on the same SAMs indicate that the ALD metal precursor plays a role in blocking ability. However, as each ALD process involves multiple aspects that could degrade SAM layers and otherwise affect nucleation (temperature, metal precursor, water exposure), determining the individual cause of the selectivity difference requires the isolation of each of these variables. To further investigate this concept, samples of each of the C6, C12, and C18 SAMs on Cu were placed in the ALD chamber and subjected to either elevated temperature (under N_2_ flow) or elevated temperature under N_2_ flow with the addition of H_2_O, TMA, or TDMAH. Each independent experiment was conducted as if a normal ALD process was occurring while omitting the other deposition steps to ensure the total time in the chamber (at the stated temperature) was identical, and all experiments were repeated at 100 °C, 140 °C, and 180 °C. In this experiment, “As-Prepared” refers to SAMs on Cu that were not subjected to ALD processing and thus represent control samples.

Sulfur and carbon atomic percentages were quantified to test the behavior of both the sulfur headgroup and the carbon chain tail group during these individual ALD steps. For experiments at 100 and 140 °C, sulfur percentages decreased as SAM chain length increased (Figure 3a,b), which may be due to some contribution from attenuation of the XPS signal through the thicker SAMs. Carbon percentages tended to increase monotonically with the SAM chain length (Figure 3d,e,f). These relative trends of sulfur and carbon percentages between SAM chain lengths remained generally consistent across temperatures and isolated ALD process variables.

**Figure 3:**
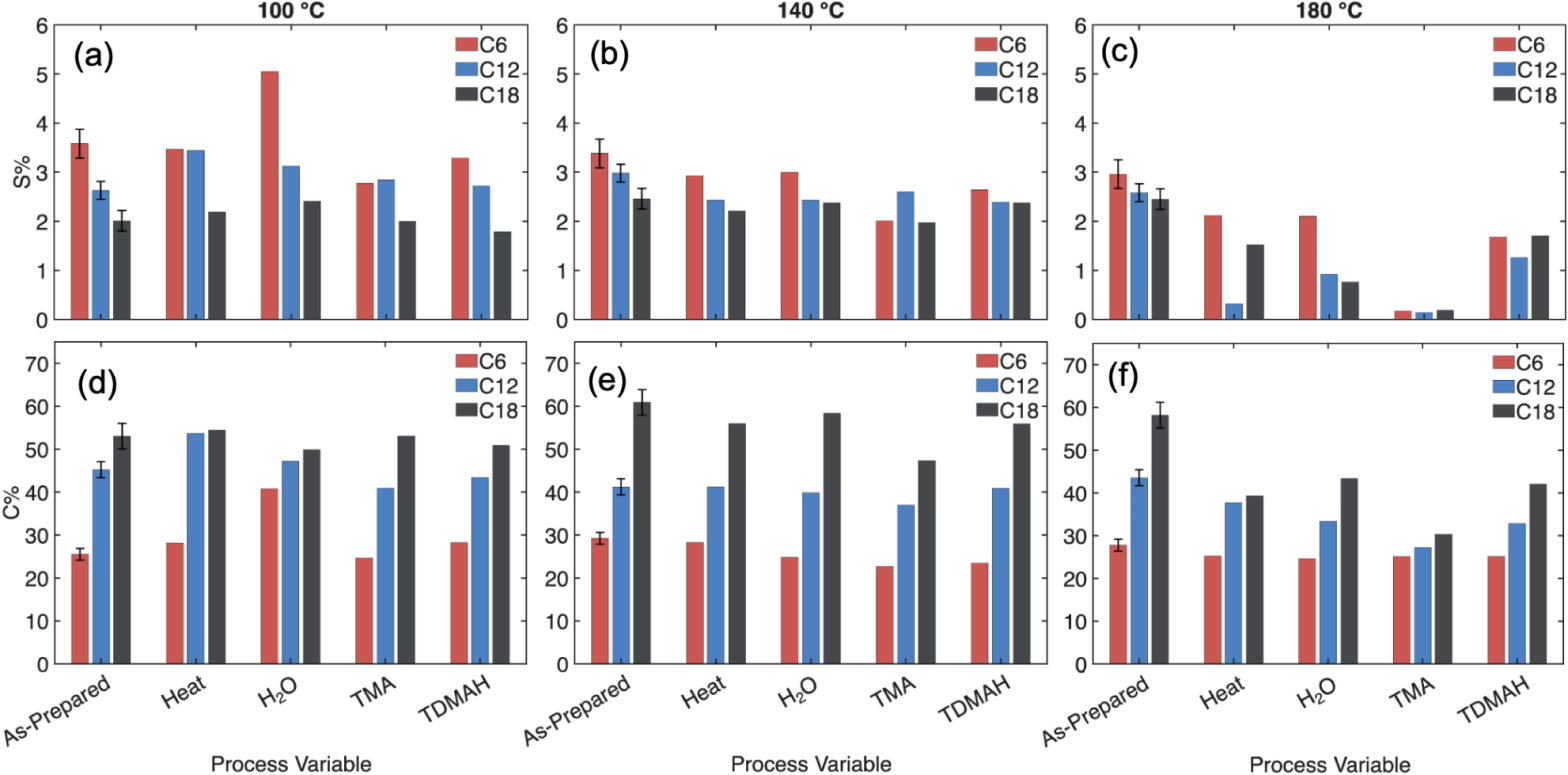
Sulfur atomic percentages (a, b, c) and carbon atomic percentages (d, e, f) for 6, 12, and 18 carbon chain length alkanethiol SAMs exposed to isolated ALD process variables: temperature alone, temperature and H_2_O exposure, temperature and TMA exposure, and temperature and TDMAH exposure. As-prepared data are for SAMs that were not subjected to any ALD processing.

Evaluating the sulfur and carbon percentage values across ALD process variables revealed the impact of isolated ALD steps on the SAMs. For each chain length, processing temperatures of 100 and 140 °C yielded consistent values across process steps, aligning closely with the “As-Prepared” samples (Figure 3a,b,d,e). For example, sulfur percentages for the C18 SAM remained between 1.5% and 2.5% across all isolated ALD variables at both 100 and 140 °C (Figure 3a,b). Such consistency was not exhibited for all SAMs—we note for example the elevated sulfur content for the C6 SAM for the “H_2_O” variable at 100 °C. Isolated variances in sulfur percentage may have been due to alkanethiol molecules adsorbed on top of the SAM surface, raising sulfur values by a few percent. Overall, even with some outliers, the general consistency across parameters and temperatures indicates that the ALD process steps at these temperatures did not significantly alter or impact the chemistry of the SAM layers. Stable SAM characteristics at 100 and 140 °C align with the previously discussed findings on sulfur retention up to 140 °C (Figure 1c, 1d) and higher selectivity for 15 cycles of ALD at 140 °C (Figure 2a, 2c).

At a process temperature of 180 °C, carbon content increased with chain length as it did at lower temperatures, though there was less carbon overall for the C18 SAMs as well as for the C12 SAMs subjected to TMA and TDMAH exposures. Additionally, at 180 °C, sulfur values and trends differed markedly from those at 100 and 140 °C, with variations among ALD variables. Exposure to each of the variables led to some decrease in the percent sulfur observed, but most notable was TMA exposure, which nearly eliminated the entire sulfur signal for all three chain lengths (Figure 3c). The corresponding carbon percentage for samples exposed to only TMA (Figure 3f) did not show as significant a drop, although they did show a decrease for C12 and C18 SAMs when compared to lower temperature and As-Prepared samples. Such loss of sulfur paired with some carbon loss may indicate SAM desorption, wherein the sulfur–copper bond is broken and the alkanethiol molecule detaches from the substrate. However, as some carbon does still remain, it is also possible that some of the S–C alkanethiol bonds were broken, leaving alkyl chain fragments on the surface. Alternatively, the remaining carbon signal could be due to the presence of adventitious carbon. We also acknowledge a degree of individual sample variability in these data, as isolated exposure data represent individual measurements. Based on the substantial loss of S observed during exposure to solely TMA compared to other process variables, it is likely that the TMA precursor plays a more significant role in SAM destabilization and drops in selectivity than the other process variables, particularly when compared to SAMs exposed to solely TDMAH. Both sterics and reactivity have been shown to influence ALD behavior,^47^ and smaller and more reactive precursors, like TMA, have been shown to penetrate through the blocking layer or react with the blocking layer surface.^48^ Specific studies comparing aluminum precursors showed that TMA is blocked less effectively than bulkier equivalents such as triethyl aluminum.^37,49^ The decrease in sulfur percentage associated with TMA exposure may explain the lower selectivity of alumina grown with TMA when compared to hafnia grown with TDMAH, particularly at 180 °C (Figure 2b, 2d). TDMAH is a bulkier molecule, and the steric hindrance may explain why hafnia deposition is blocked more effectively than the alumina analog using TMA. Additionally, analysis of the copper Auger spectra revealed a change in peak shape for TMA exposure at 180 °C on the C6 SAM (Figure S2) compared to other exposure conditions at that temperature. Specifically, a decrease was observed in the intensity of the Cu_2_O peak (also overlapping with the Cu_2_S peak), which suggests a change in oxidation state.^50,51^ However, such a change was not observed for the other chain lengths, despite these still showing significant sulfur loss. Finally, while this study has shown how isolated ALD parameters impact SAM blocking behavior and surface elemental composition, a concrete statement of the mechanisms by which these parameters exert their impact cannot be made beyond conclusions based on prior literature. We recommend such a line of investigation for future studies, in particular focusing on the mechanisms by which the TMA molecule causes drops in sulfur percentage and blocking ability of SAMs at high temperature, as well as investigating the association between copper Auger spectra and Cu–S bond changes during ALD processing.

## Conclusion

We have investigated the use of alkanethiol SAMs as blocking layers for AS-ALD of alumina and hafnia, focusing on the influence of alkanethiol chain length (*C* = 6, 12, 18) on SAM stability and blocking ability. Additionally, ALD process variables (temperature, oxidant, metal precursor) were isolated to determine their individual impact on ASD. Selectivity toward hafnia deposition increased with chain length, but for selectivity toward alumina, no clear chain-length dependence emerged. The alkanethiol SAMs were more selective toward hafnia than alumina, and more selective at 140 °C than 180 °C. Sulfur and carbon XPS analysis revealed temperature-dependent SAM degradation, with significant amounts of sulfur loss at 180 °C, suggesting SAM desorption as a primary mechanism of blocking layer degradation. Among isolated ALD process variables, TMA exposure demonstrated the most significant effect, leading to a near elimination of the sulfur signal for all chain lengths at 180 °C. Such an effect may be due to the small size and high reactivity of the TMA molecule in contrast to the bulkier TDMAH, potentially enabling penetration of the precursor to the SAM sulfur–copper bond. We recommend future studies to more specifically interrogate the mechanisms of SAM destabilization. These findings highlight the interplay of SAM structure, ALD precursor chemistry and temperature in ASD, and the potential tunability of these parameters to create optimized ASD conditions for diverse precursor/blocking layer systems.

## Supplementary Material



## Notes

Figure S1: Precursor and monolayer chemistries; Figure S2: Copper Auger peaks (Cu LMM) for samples from Figure 3, with isolated exposure to different ALD parameters (PDF)

## #

Department of Materials Science and Engineering, Massachusetts Institute of Technology, Cambridge, Massachusetts, 02139, United States
