# Supplementary material for: Stability of Alkanethiol Self-Assembled Monolayers of Varied Chain Lengths for Area-Selective Atomic Layer Deposition
Source: Langmuir. 2026 Jul 15;42(29):21049–57. doi: 10.1021/acs.langmuir.6c01484 (PMC13421965; doi:10.1021/acs.langmuir.6c01484)
Supplement: Supplementary file 1 [file la6c01484_si_001.pdf]

Stability of Alkanethiol Self-Assembled Monolayers of Varied Chain Lengths for Area-Selective Atomic Layer Deposition

Henry Price[1,†], Vamseedhara Vemuri[1], Michelle M. Paquette[2], Nicholas C. Strandwitz[1,]*

[1] Department of Materials Science and Engineering and Institute for Functional Materials and Devices, Lehigh University, Bethlehem, Pennsylvania 18015, USA

[2] Missouri Institute for Defense & Energy and Division of Energy, Matter & Systems, University of Missouri Kansas City, Kansas City, Missouri 64110, USA

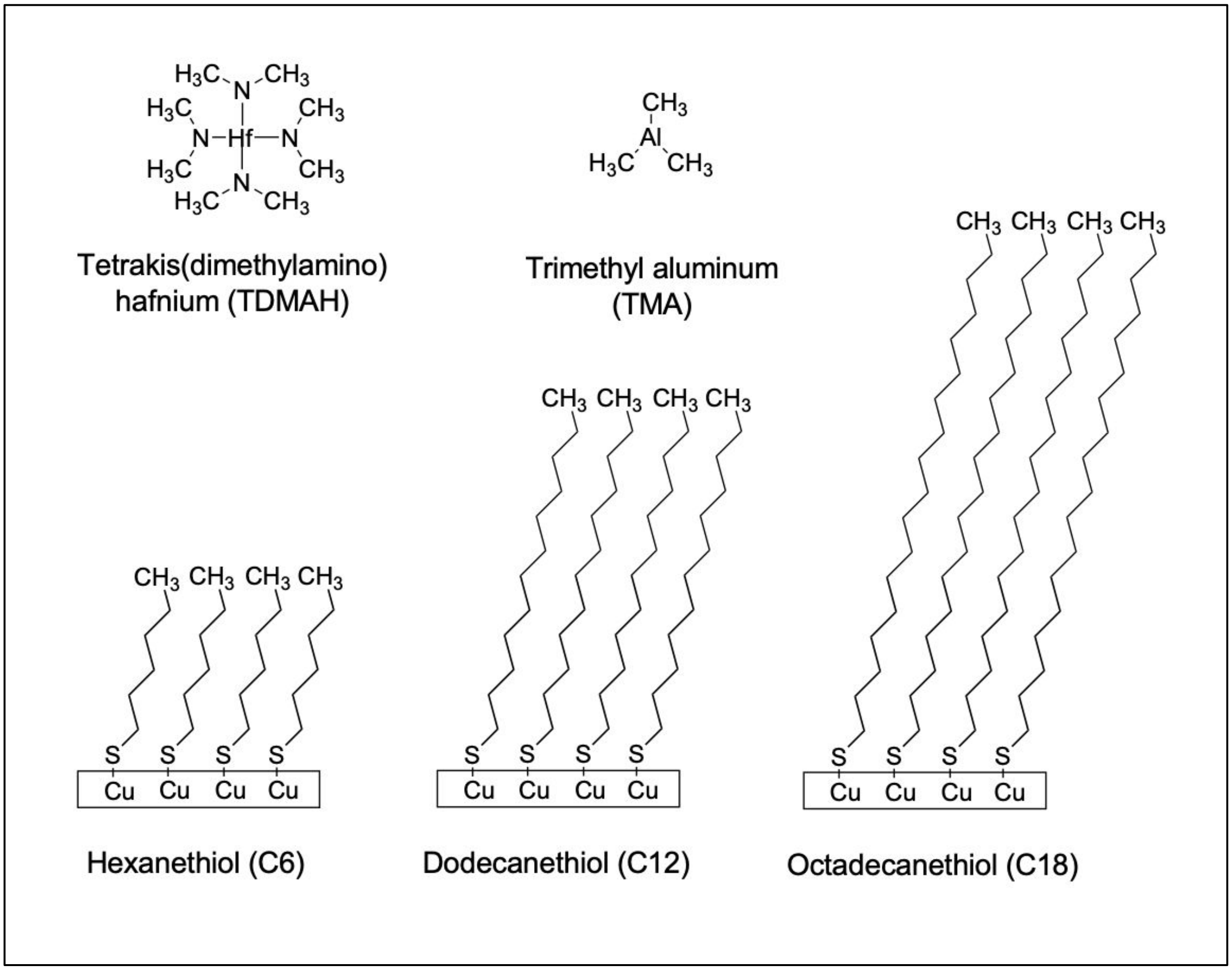

Figure S1. ALD metal precursors and self-assembled monolayer chemistries used in this study.

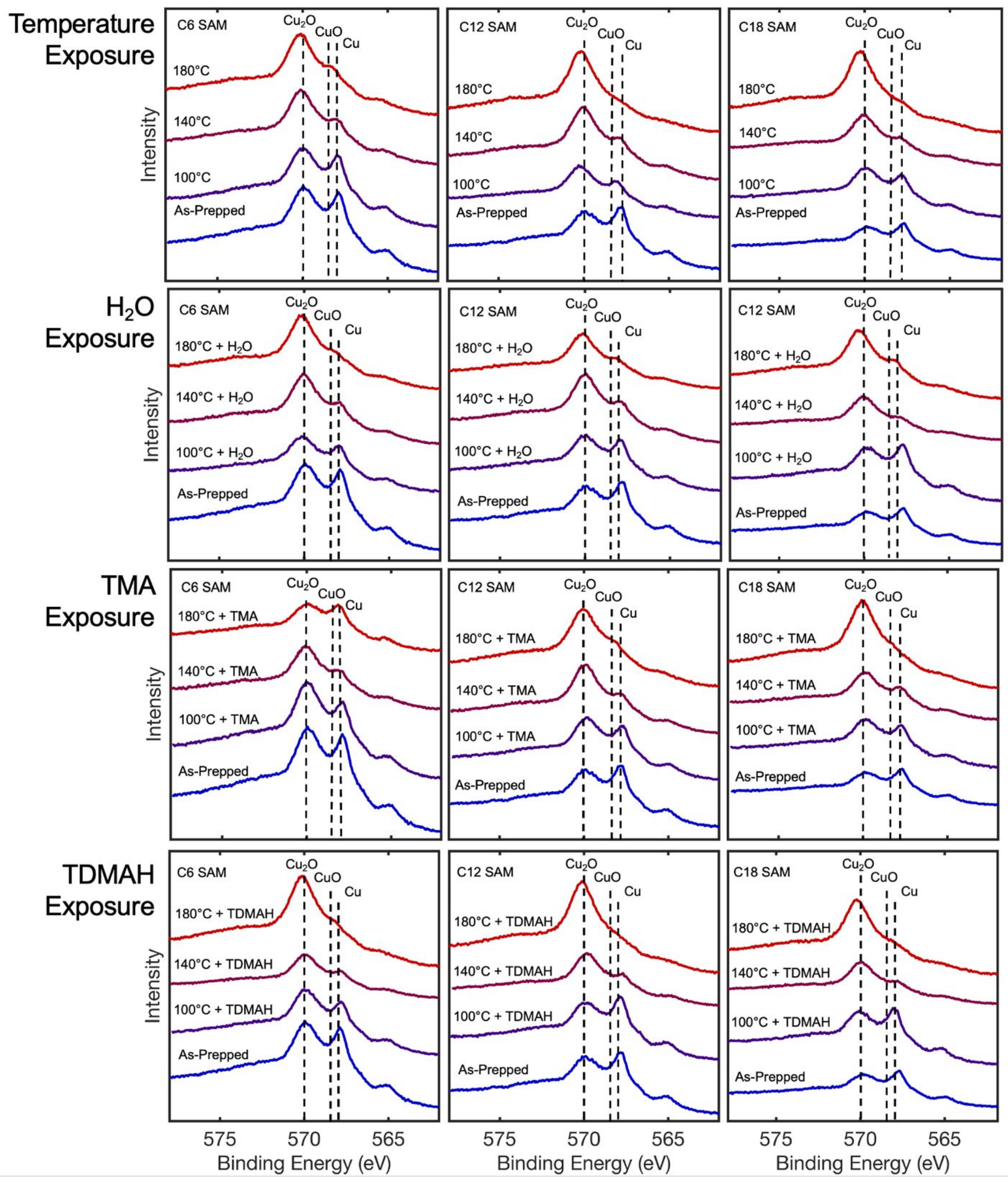

Figure S2. Copper auger peaks (Cu LMM) for samples from Figure 3, with isolated exposure to different ALD parameters
